# MALDI-2 Mass Spectrometry for Synthetic Polymer Analysis

**DOI:** 10.1021/acs.macromol.3c01401

**Published:** 2023-09-22

**Authors:** Lidia Molina-Millán, Aljoscha Körber, Bryn Flinders, Berta Cillero-Pastor, Eva Cuypers, Ron M. A. Heeren

**Affiliations:** † The Maastricht MultiModal Molecular Imaging Institute (M4i), Division of Imaging Mass Spectrometry, Maastricht University, Universiteitssingel 50, 6229 ER Maastricht, The Netherlands; ‡ The MERLN Institute for Technology-Inspired Regenerative Medicine, Department of Cell Biology-Inspired Tissue Engineering, Maastricht University, Universiteitssingel 40, 6229 ER Maastricht, The Netherlands

## Abstract

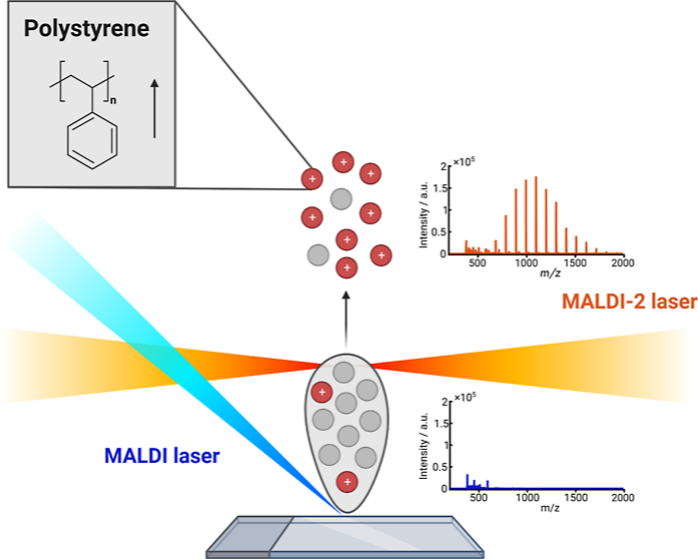

Synthetic polymers are ubiquitous in daily life, and their properties offer diverse benefits in numerous applications. However, synthetic polymers also present an increasing environmental burden through their improper disposal and subsequent degradation into secondary micro- and nanoparticles (MNPs). These MNPs accumulate in soil and water environments and can ultimately end up in the food chain, resulting in potential health risks. Matrix-assisted laser desorption/ionization mass spectrometry imaging (MALDI MSI) has the potential to study localized biological or toxicological changes in organisms exposed to MNPs. Here, we investigate whether MALDI-2 postionization can provide a sensitivity enhancement in polymer analysis that could contribute to the study of MNPs. We evaluated the effect of MALDI-2 by comparing MALDI and MALDI-2 ion yields from polyethyleneglycol (PEG), polypropylene glycol (PPG), polytetrahydrofuran (PTHF), nylon-6, and polystyrene (PS). MALDI-2 caused a signal enhancement of the protonated species for PEG, PPG, PTHF, and nylon-6. PS, by contrast, preferentially formed radical ions, which we attribute to direct resonance-enhanced multiphoton ionization (REMPI). REMPI of PS led to an improvement in sensitivity by several orders of magnitude, even without cationizing salts. The improved sensitivity demonstrated by MALDI-2 for all polymers tested highlights its potential for studying the distribution of certain classes of polymers in biological systems.

## Introduction

Synthetic polymers are found everywhere in daily life. We benefit from their technical properties in electronics, household appliances, construction materials, coatings, packaging, clothing, personal protection, homeland security, and food science, and the list of applications gets longer and longer. Their sustainable use through recycling and upcycling of our waste streams has increased in recent years.^1^ Still, global plastic production is tremendously increasing, which translates into millions of tons of plastic waste being generated. Between 1950 and 2017, 76% of global plastic waste was discarded and ultimately ended up in landfills, dumps, or the natural environment.^2,3^ There, the polymeric materials are exposed to weathering, and together with wear and tear, smaller and smaller micro- and nanoparticles (MNPs) are formed. MNPs can easily accumulate in soil,^4^ the water environment,^5^ the food chain,^6^ and even in humans.^7^ Several articles have already associated these polymer particles with potential health risks.^8,9^ Multiple studies have indicated that a comprehensive analysis of MNPs is essential for the study of their biological toxicity.^10–12^

MNPs have various chemical compositions and physical properties. It is therefore essential to measure and consider all relevant characteristics of MNPs, including source, size, shape, surface charge, surface chemistry, and exposure concentration when we attempt to understand the interaction of these contaminants with biological systems.^10^

The study of MNPs thus poses several analytical challenges. First, MNPs are so small that they challenge the sensitivity of common analytical approaches. In some cases, they can be visualized with light or electron microscopy,^10^ but these techniques provide no information on the chemical identity of the polymers and additives of each MNP. Second, MNPs exhibit a plethora of chemical compositions, which is further increased by chemical degradation and leaching in the environment.^13^ Optical microspectroscopy, such as IR and Raman, is useful to identify the polymer class of MNPs, but lacks the ability to elucidate the more complex composition of polymers, including structure, additives, end group identity, polymerization degree, and molar mass distribution. Third, MNPs end up in a wide variety of matrices, ranging from “simple” aquatic matrices to more complex ones, for instance, prepared food, soil, plants, or complete biological systems such as insects, animals, or even human samples.^7,14–16^

The question then arises: what analytical methods are appropriate for the study of MNPs in situ? One technology that can unravel the chemical complexity of synthetic polymers is multidimensional mass spectrometry (MS).^17^ Soft ionization methods, such as electrospray ionization (ESI) and matrix-assisted laser desorption/ionization (MALDI), are employed for synthetic polymer analysis and provide information on a wide variety of intact macromolecules as well as low-molecular-weight additives. Bulk polymer analysis is often performed by dissolving a polymer in a pure solvent followed by chromatographic separation combined with electrospray-based MS.^18^ However, the sample preparation routine described above implies losing most spatial information and prevents the correlation of MNPs with local toxicological changes in biological matrices. Furthermore, ESI generates multiply charged species and can result in complex spectral patterns that require high-resolution MS to resolve and identify its constituents.^19–22^

In comparison, MALDI circumvents the disadvantages of ESI-MS for synthetic polymer analysis. In particular, MALDI-TOF MS is capable of determining molecular weight distributions,^23^ end-group compositions,^8^ and polymer dispersity.^24^ MALDI commonly provides singly charged species through protonation or metal cationization, resulting in reduced spectral complexity compared to that of ESI. In most studies, the polymer is dissolved and mixed with a matrix prior to analysis. However, MALDI MS can also be performed locally to study the spatial distributions in complex matrices as its laser can be focused on micron-size spots.^25^ This feature is exploited extensively in the field of MALDI MS imaging (MSI) for biological tissue^26^ and makes it suitable for the in situ analysis of MNPs in biological systems. The use of MALDI MSI to study the local toxicity of drugs and their metabolites has already been demonstrated.^27^ Nonetheless, not many MALDI MSI studies on MNPs in tissue have been conducted, which we attribute to the insufficiently low sensitivity of MALDI MSI. More precisely, only 0.1–0.01% of all available molecules are typically ionized with MALDI,^29^ and this number can be even lower for molecules without easily ionizing functional groups and due to ion suppression caused by tissue heterogeneity.^30^ An additional aggravating factor is the small size of the MNPs, which limits the amount of available polymer molecules that can be ionized. For instance, one study employed MALDI MSI to study the uptake of high-density polyethylene microplastics into quagga mussels but lacked the sensitivity to detect repeating polymer peaks.^28^

MALDI-2, in which a second pulsed laser (λ < 280 nm) orthogonally intercepts the primary MALDI plume, can boost the ionization efficiency of MALDI by 2–3 orders of magnitude.^31^ Pressures between 1.5 and 10 mbar are typically used in MALDI-2 experiments,^32^ but ambient conditions also have been implemented.^33^ The exact MALDI-2 mechanism remains under discussion, but according to current studies^32^ MALDI-2 is thought to promote the ionization of excess matrix neutrals in the MALDI plume. In a gas-phase ion–molecule reaction, neutral analyte molecules can react with the additional charges that have become available, resulting in an overall increase in ionization efficiency. Moreover, the higher ion yields achieved with MALDI-2 demonstrate the practicality of this technique in MSI experiments when working with small pixel sizes.^34^

MALDI-2 has not yet been applied to the study of synthetic polymers. We hypothesize that an enhanced ionization efficiency through MALDI-2 will allow the analysis of certain polymers with an increased sensitivity. Additionally, it could pave the way for analyzing MNPs in tissue with MALDI-2 MSI as the increased sensitivity benefits spatial resolution.^34^ In this article, we study the effect of MALDI-2 in the analysis of three polymer families: polyethers (polyethylene glycol, polypropylene glycol, and polytetrahydrofuran), polyamides (nylon-6), and hydrocarbons (polystyrene). Some of these polymers, such as PS, are commonly found in the marine environment as MNPs, while others are used as water-soluble food additives. We compare and contrast normal MALDI with MALDI-2 with respect to polymeric distribution and also examine the respective spectral appearance of the polymer in light of the fundamental ionization processes. By doing so, this study aims to provide more insight into the application of MALDI(-2) for in situ polymer analysis.

## Materials and Methods

### Reagents and Solvents

Polyethylene glycol (PEG, *M*_w_ ∼ 1500 Da) was supplied by Fluka Chemie GmbH (Buchs, Switzerland). Polypropylene glycol (PPG, *M*_w_ ∼ 1010 Da, *M*_w_/*M*_n_ 1.04) was purchased from PSS Polymer Standards Service GmbH (Mainz, Germany). Polytetrahydrofuran of *M*_w_ ∼ 1000 Da and *M*_w_ ∼ 1400 Da (denoted as PTHF1000 and PTHF1400, respectively) was obtained from Royal DSM N.V. (Heerlen, The Netherlands). Nylon-6 was purchased from Scientific Polymer Products Inc. (*M*_w_ ∼ 25,000 Da, Oregon, USA). Polystyrene (PS, *M*_w_ ∼ 1300 Da, *M*_w_/*M*_n_ 1.10) was purchased from Thermo Fisher Scientific GmbH (Kandel, Germany). Polybutylene terephthalate (PBT, *M*_w_ ∼ 16,150 Da). ULC-MS-grade methanol (MeOH), ethanol (EtOH), and *n*-hexane were acquired from Biosolve B.V. (Valkenswaard, The Netherlands). Tetrahydrofuran (THF) and hexafluoroisopropanol (HFIP, ≥99% purity) were supplied by Sigma-Aldrich Chemie B.V. (Zwijndrecht, The Netherlands). All organic solvents were used without further purification. 2,5-Dihydroxybenzoic acid (DHB, 98% purity), dithranol (DT, ≥90% purity), 2′,4′,6′-trihydroxyacetophenone monohydrate (THAP, ≥99.5% purity), silver trifluoroacetate salt (AgTFA, 98% purity), and trifluoroacetic acid (TFA, ≥99% purity) were purchased from Sigma-Aldrich.

### Sample Preparation

#### Polyethers

A PEG stock solution was prepared (1 mg/mL, MeOH) and mixed with a DHB matrix solution (20 mg/mL, MeOH) at a volume ratio of 1:5. The mixture was diluted with MeOH 1:1 (v/v) to a final PEG concentration of 0.083 mg/mL. The mixture was sprayed manually onto a conductive indium tin oxide (ITO)-coated glass slide (Delta Technologies Ltd., Loveland, USA) with a thin-layer chromatography (TLC) spraying device (Sigma-Aldrich). In total, 15 layers were applied. PPG, PTHF1000, and PTHF1400 were prepared following the same procedure as that for PEG.

#### Nylon-6

A nylon stock solution was prepared (1 mg/mL, HFIP) and mixed with a DHB matrix solution (20 mg/mL, MeOH) at a ratio of 1:5 (v/v). The mixture was diluted with MeOH 1:1 (v/v) to a final concentration of 0.083 mg/mL. Next, the diluted solution was sprayed onto an ITO slide with a TLC sprayer device (15 layers).

#### Polystyrene

A PS stock solution (0.5 mg/mL, THF) was added to three different vials. One vial contained the polymer solution with additional THF solvent. The second vial contained the polymer and matrix mixture at a ratio of 1:4 (v/v). The third vial contained PS, DT, and AgTFA in a ratio of 1:4:1 (v/v). All three vials had a final PS concentration of 0.083 mg/mL. The DT solution was filtered with a 25 mm, 0.45 μm PTFE filter (VWR LLC, Leuven, Belgium) before mixing. The mixtures were sprayed separately onto an ITO slide with a TLC device. Fifteen layers were applied per mixture. As a result, one-third of the slide was covered with each of the prepared solutions.

All slides were air-dried prior to analysis.

#### Polybutylene Terephthalate

PBT was dissolved with HFIP to a final concentration of 0.5 mg/mL. 0.5 μL of THAP (20 mg/mL, THF) was spotted and air-dried on a MTP 384 ground steel target plate (Bruker Daltonics GmbH & Co. KG, Bremen, Germany). 0.5 μL of PBT solution was deposited on top and allowed to dry. This protocol was adapted from Lou et al.^50^

### MS Measurements

A timsTOF fleX (Bruker Daltonics) instrument equipped with two Nd/YAG lasers at fixed wavelengths of 355 and 266 nm for MALDI and MALDI-2, respectively, was used for all experiments. All spectra were collected in positive-ion mode between 200 and 3000 *m*/*z*. Calibration of the *m*/*z* scale was performed with red phosphorus in positive-ion mode prior to the acquisition. FlexControl and FlexImaging (Bruker Daltonics) software were used for MSI data acquisition. Each polymer standard was sequentially analyzed with MALDI-2 and conventional MALDI. Unless specified otherwise, all measurements were performed with the following settings: 1000 Hz repetition rate, 50 laser shots per pixel, and 75% of the laser power. Data were acquired by setting up a rectangular scan area of 5 mm^2^ and using a 100 μm step size. MALDI-2 was performed with the following parameters: 266 nm wavelength, a pulse energy of ∼30 μJ, and a pulse delay time (*t*_delay_) of 23 μs with respect to the firing of the MALDI laser. For the analysis of PS, the settings for the primary laser were: 1000 Hz repetition rate, 250 laser shots, and 95% laser power. In this case, the *t*_delay_ of the MALDI-2 laser was set to 13 μs. For PBT, only spectra were acquired with MALDI and MALDI-2. The primary laser was set to 1000 Hz, 10,000 shots, 55% power, and 30 μm spot size, whereas the *t*_delay_ of the second laser was 10 μs to obtain PBT data.

Additional experiments were performed by modifying the trigger delay time of the MALDI-2 laser. Data were acquired at delay times of 13, 23, 33, 43, 53, and 63 μs between the first and second laser pulse. For these experiments, we used the same ITO slide sprayed with 15 layers of PS and DT mixture (see above). We also tested an ITO slide sprayed with 10 layers of a PPG (1 mg/mL, MeOH) and DHB (20 mg/mL, MeOH) mixture [1:5 (v/v)].

### UV–Vis Measurements

PS solution (0.1 mg/mL, THF) and PBT (0.0083 mg/mL, HFIP) solutions were measured with a quartz cuvette (Hellma GmbH & Co. KG, Müllheim, Germany) in an Agilent Cary 60 UV–vis spectrophotometer (Agilent Technologies Netherlands B.V., Amstelveen, The Netherlands). THF and HFIP were measured, respectively, as a blank for baseline subtraction. The absorbance spectrum was recorded in the 200–600 nm region. Agilent Cary WinUV (Agilent Technologies) software was used for data acquisition.

### Data Analysis

Mass spectra were opened with SCiLS Lab (Bruker Daltonics) and exported without further processing as .csv files. These files were then loaded into MATLAB R2020a (MathWorks Inc., Natick, USA), where replicate spectra were averaged and polymer peaks were identified automatically by comparing their *m*/*z* with calculated monoisotopic values. Height intensities and standard deviations of identified polymer peaks were used to calculate MALDI-2/MALDI ion yield ratios for each peak. For further comparisons, we also simulated isotopic distributions with IsoPro 3.1 freeware. All spectra were plotted with MATLAB.

## Results and Discussion

In this work, we employed MALDI-2 and MALDI to analyze PEG, PPG, PTHF, nylon-6, and PS standards in positive-ion mode. We spray-coated all polymers instead of depositing dried droplets to reduce sample heterogeneity.^35^

### Polyethers and Nylon-6

**Figure 1:**
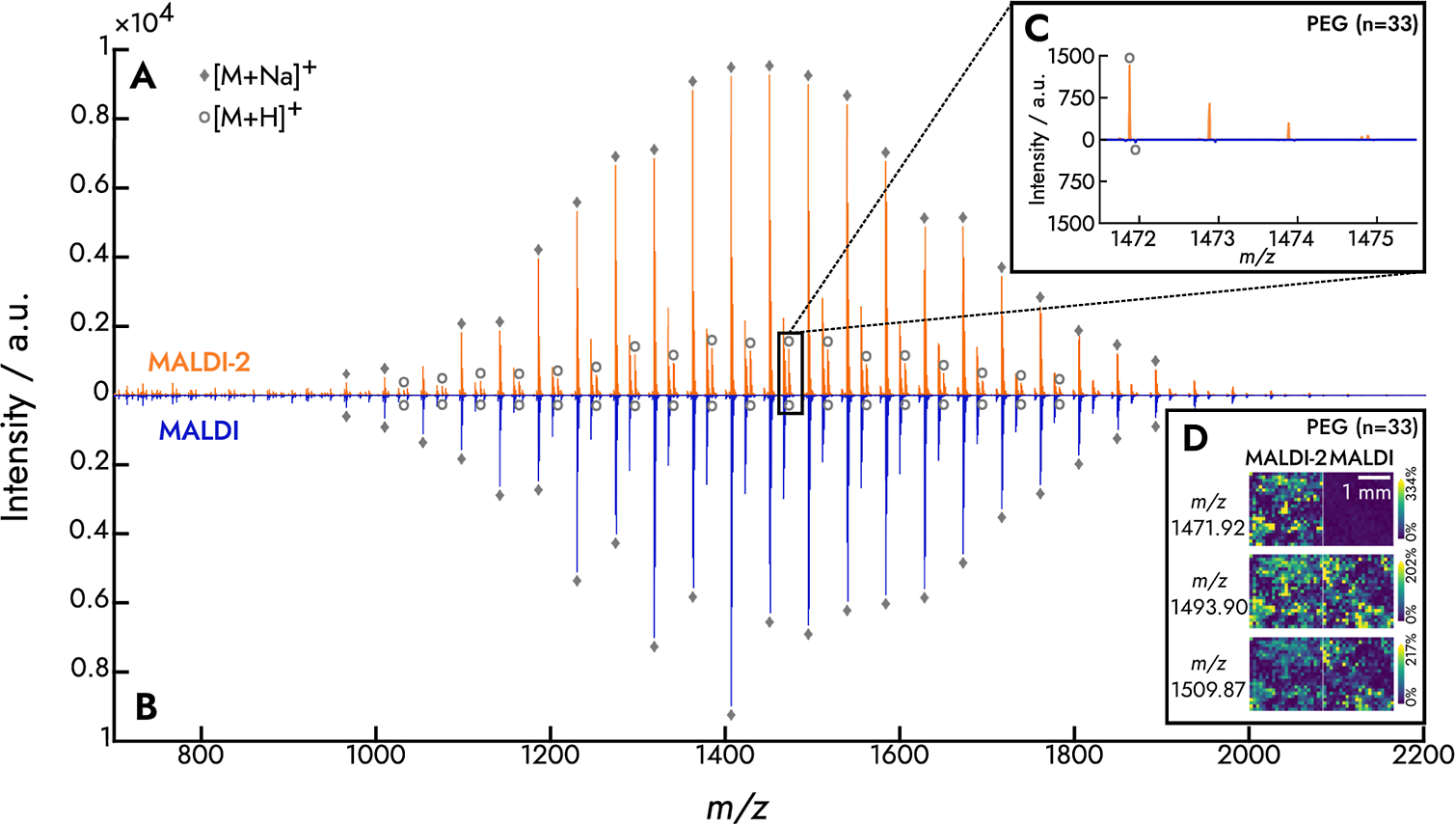
MALDI-2 and MALDI spectra of PEG. Both MALDI-2 (A) and MALDI (B) spectra are dominated by sodiated (gray ⧫) adducts. MALDI-2 resulted in a more than 10-fold increase in the protonated ion signal (gray ○), as exemplified in a magnified inset (C). Meanwhile, the intensities of sodiated PEG ions remained constant. MALDI-2 and MALDI MSI images of PEG (D) illustrate the effect of the MALDI-2 laser on protonated, sodiated, and potassiated species.

We obtained similar results for PEG, PPG, PTHF1000, PTHF1400, and nylon-6 in MALDI and MALDI-2. The ionization yields of PEG with MALDI-2 (orange) and MALDI (blue) are compared in detail in Figures 1 and S1. Spectral data of PPG, PTHF1000, PTHF14000, and nylon-6 are provided in Figures S2 and S3.

The lists of *m*/*z* values, peak intensities, identities, ion types, and mass errors corresponding to Figure 1 are provided in the Supporting Information.

With MALDI-2 (Figure 1A), the spectrum for PEG comprised [M + Na]^+^, [M + K]^+^, and [M + H]^+^ ions. No radical cations were detected for PEG. The difference of 44 *m*/*z* between two consecutive peaks of the same species corresponds to the repeating unit, [C_2_H_4_O]. PEG sodium adducts are predominant in the mass spectrum since the alkali ion affinity of PEG is higher than its proton affinity.^36^

The signal intensities for [M + Na]^+^ and [M + K]^+^ ions (annotation not shown for the latter) were comparable in both MALDI-2 and MALDI (Figure 1A,B), arguing that MALDI-2 had no effect on alkali species. The ion signal of [M + Na]^+^ and [M + K]^+^ species also remained constant for PPG, PTHF1000, PTHF1400, and nylon-6 polymers. Prior studies have shown similar trends for [M + Na]^+^ and [M + K]^+^ ions.^31,32^

Protonated species of PEG increase, in contrast, with MALDI-2 by at least an order of magnitude. For instance, the [M + H]^+^ ion at *m*/*z* 1471.92 (*n* = 33) was detected with a 29-fold higher signal using MALDI-2 (Figure 1C). Protonated species of PPG, PTHF1000, PTHF1400, and nylon-6 increased as well, reaching a 140-fold gain compared to regular MALDI (Figures S2 and S3). Nylon-6 showed the smallest MALDI-2 improvement for protonated species (∼1.3-fold), which we attribute to its higher polymer weight distribution (*M*_w_ ∼ 25,000 Da). Moreover, analyzing nylon-6 with MALDI is challenging due to its low solubility in most solvents. The solvent used here, HFIP, is likely too harsh for imaging biological tissues when it is used during the matrix application.

**Scheme 1:**
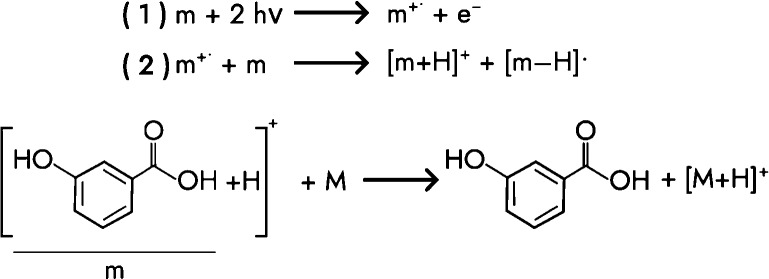
Tentative MALDI-2 Mechanism Involving Sequential Ionization Reactions (adapted from Potthoff et al.^32^)

The results in the protonated species are in agreement with earlier studies in which the signal of these species is enhanced by the second laser.^37–40^ The boost in ion yield for protonated species can be explained by a mechanism proposed for MALDI-2 by Soltwisch et al., in which the matrix (*m*) is ionized by resonance-enhanced multiphoton ionization (REMPI), causing the formation of radical matrix cations (Scheme 1).^31,32,39^ These radical ions abstract a proton from another matrix molecule, which, given their acidity (e.g., DHB), they then transfer to neutral analyte molecules (M).

The discussed MALDI-2 effect in the protonated, sodiated, and potassiated forms is also illustrated for PEG in Figure 1D.

While ionization was enhanced by MALDI-2 for these five polymers, the degree of enhancement for protonated species varied by mass and degree of polymerization of the polymers (Figure S4). We considered several explanations but were not able to pinpoint this observation to a single phenomenon. Thermodynamics are unlikely to have a strong influence, given the energetically activated environment of the MALDI plume. Increased polymer folding leading to smaller collision cross sections, better steric protection, and thus less protonation seems improbable as well because polymer folding (as found for PEG) is mainly observed only for multiply charged ions.^41^ Concentration-driven kinetics might be a more plausible explanation. However, the center (where the polymer is most abundant) of each polymer distribution should then exhibit the largest signal enhancement. We did not observe such a trend (Figure S5), possibly explained by the variance in our measurements that did not allow us to discern clearly this trend. Furthermore, the observed polymer ion distributions might not accurately reflect the polymer distribution in the part of the MALDI plume that is irradiated with the second laser. For instance, the speed of polymers in the gas phase might vary with their mass, leading to a dependence between MALDI-2 ion yield and the delay time between the firing of the first and the second laser. Therefore, we varied the delay time for the measurement of PPG (Figure S6) and PEG (not shown) but did not observe preferential ionization of heavier polymers with delay time. Varying the delay time only resulted in an increase in overall ionization efficiency. MALDI-2 was most efficient at delay times between 23 and 33 μs, which differs from the optimum delay time of our instrument for lipids and metabolites, which is ∼13 μs. Our investigation into the different ionization efficiency of MALDI-2 with mass and degree of polymerization thus remains inconclusive and likely is caused not by one but several effects.

### Polystyrene

**Figure 2:**
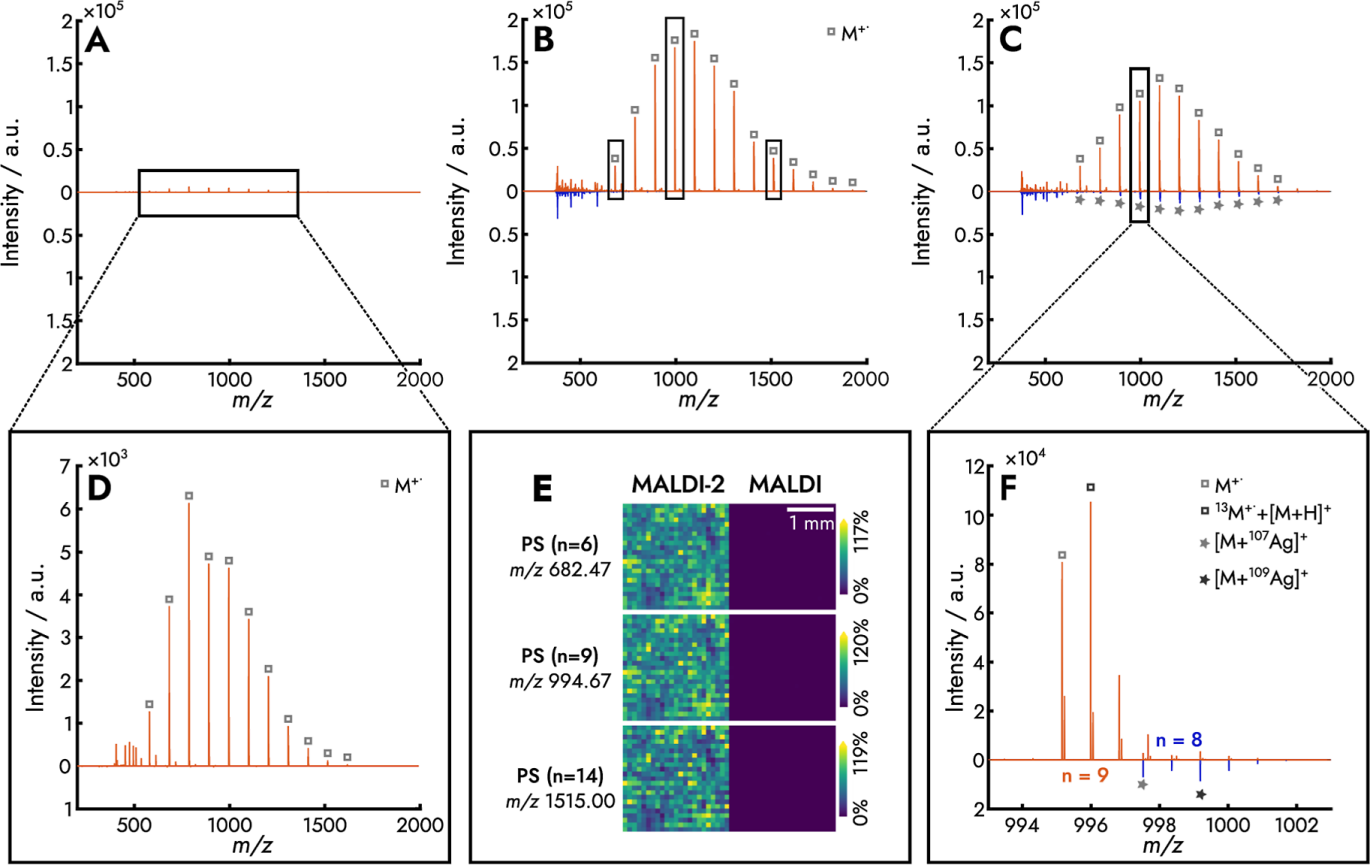
Effect of MALDI-2 on PS in three conditions: without matrix (A), with DT matrix (B), and with the DT matrix and AgTFA cationizing agent (C). LDI-PI (D) promotes the formation of radical ions with a shift in the polymeric distribution toward lower *m*/*z* values. The absence of a matrix hinders the desorption of analyte molecules and hence the detection of larger PS oligomers. When adding matrix (B), we obtain higher ion intensities and a more representative *M*_w_ distribution of PS with MALDI-2. MALDI-2 and MALDI MSI images (E) show the preferential formation of radical ions by MALDI-2. Adding AgTFA (C) enhances the ionization of PS by MALDI, but still the radical ions formed with MALDI-2 are more intense. The magnification of the isotopic fine structure of M^+•^ and [M + Ag]^+^ ions (F) illustrates the different ionization mechanisms with conventional MALDI and MALDI-2.

Unlike the polymers discussed so far, PS yielded orders-of-magnitude stronger signal enhancement with MALDI-2 (Figure 2). We compared the ionization efficiency of PS in three different conditions: polymer (Figure 2A), polymer mixed with the DT matrix (Figure 2B), and polymer mixed with the DT matrix and AgTFA (Figure 2C). In all conditions, the delay time was 13 μs between the first and the second laser, as only small improvements in ion yield were seen with delay time (Figure S7).

Figure 2A shows laser desorption postionization, LDI-PI (orange, top), and laser desorption ionization, LDI (blue, bottom), for PS. The magnified spectrum (Figure 2D) reveals that radical species (M^+•^) were observed with LDI-PI whereas no signal was obtained with LDI. The maximum peak of the PS distribution was at *m*/*z* 786.52 (*n* = 7), which is a lower *m*/*z* value than expected for PS1300. We attribute this underestimation in the *M*_w_ distribution to the higher volatility of lighter molecules. Therefore, desorption limitations should be considered when analyzing larger polymeric chains without a matrix. As PS strongly absorbs at the wavelength of the MALDI-2 laser (Figure S8), we attribute the formation of radical PS cations to direct 1 + 1 REMPI of PS.^49^ Infrared laser desorption (IR-LD) REMPI of PS has been shown before,^42^ but the mass spectra were dominated by the styrene monomer and its fragments. Larger oligomers containing more than six monomer units were not detected.

Some synthetic polymers, such as PS, typically require cationizing agents to be detected with MALDI as adducts with alkali or transition metal ions. In the case of PS, silver salts are commonly added to matrices to produce higher MALDI signals. It has been demonstrated that using AgTFA and DT produces the most sensitive and reproducible results when analyzing low-molecular-weight PS with MALDI.^43,44^ Nonetheless, we found that PS was detected with higher ion yields without AgTFA by using MALDI-2 (Figure 2B). In this case, we observed an increase in ion yield by at least 5 orders of magnitude with MALDI-2 (Figure 2B, orange, top). The boost in signal is exemplified with the M^+•^ ions at *m*/*z* 682.47, 994.67, and 1515.00, as shown in Figure 2E. The addition of the DT matrix seems to facilitate the desorption but not the ionization process, as no PS signals were detected with MALDI alone (Figure 2B, blue, bottom). We interpret that both desorption and ionization mechanisms are described best as MALD 1 + 1 REMPI.

The *M*_w_ distribution is centered at ∼1100 Da, which is more representative than the results obtained with LDI-PI. Still, MALDI(-2) underestimates the *M*_w_ stated by the manufacturer, as heavier molecules are increasingly difficult to desorb. In the future, this drawback can be bypassed by combining MALDI-2 MSI with laser capture microdissection of selected sample spots, followed by their analysis via high-performance liquid chromatography ESI-MS.

Adding AgTFA to the DT matrix and PS allows observing Ag adducts of PS with MALDI, which is consistent with previous findings (Figure 2C, blue, bottom).^44^ MALDI-2 led again to the formation of radical cations, presumably via direct REMPI (Figure 2C, orange, top), but the increase in radical ion yield was less pronounced than that without AgTFA (Figure 2B). The isotopic fine structure of PS peaks reveals a minor contribution of protonated PS ions (Figures 2F and S9). We attribute this finding to gas-phase protonation of PS via the tentative MALDI-2 mechanism (Scheme 1). The amount of protonation, however, is minor because neither DT nor PS are acidic.^45,46^

Note that the maximum of the polymer distribution observed with MALDI-2 appears to be shifted toward lower *m*/*z* compared to MALDI. This is because the polymer distribution in MALDI consists of silver adducts, while with MALDI-2, it consists of radical ions. The mass of silver (107 Da) is similar to that of a PS monomer (104 Da).

Furthermore, we also observed a decrease in the silver adduct ion yield with laser postionization. MALDI-2 more than halves the signal for silver adducts with PS, whereas, for instance, sodiated adducts of highly sodiated PEG samples did not change significantly (Figure S10). This behavior is uncommon for MALDI-2 and highlights that the mechanism involved may be specific for molecules undergoing alternative ionization pathways such as REMPI. We suspect that resonant photofragmentation might be occurring next to REMPI since PS strongly absorbs at 266 nm.

Comparing all conditions tested for PS, we demonstrated that PS can be better detected, even without silver salts using MALDI-2 (Figure 2). This finding could present an advantage for the analysis of synthetic polymers with MALDI since the addition of a cationizing agent will not be required for polymers with sufficiently high absorption at the wavelength of the second laser. In addition, prior studies reported that silver salts when used with very acidic matrices can produce spectral interferences in the analysis of low-molecular-weight polymers.^47,48^ Hence, MALDI-2 could be very beneficial for this kind of study of PS without spectral interferences. Lastly, other UV-absorbing polymers, such as those containing aromatic systems, could benefit from a MALDI-2-based analytical approach. We tested PBT and a spectral comparison revealed only a marginal improvement (Figure S11) as PBT does not absorb at 266 nm (Figure S12). In this and similar cases, using a tunable laser for MALDI-2 would allow for precise wavelength matching and hence maximum ion yield.

## Conclusions

Although MNPs pose an increasing environmental and potential health risk, there is a lack of methods to study these challenging pollutants and their interactions with tissues. MALDI MSI has the potential to analyze MNPs in situ, but it presents sensitivity limitations. In this article, we studied whether MALDI-2 postionization could provide higher sensitivity to analyze polymer MNPs. Here, we present for the first time an analysis of polymers with MALDI-2. MALDI-2 resulted in a signal increase of protonated species for polyethers (PEG, PPG, PTHF1000, and PTHF1400) by 1–2 orders of magnitude, but sodiated species are still dominant in the mass spectra. Nylon-6 behaved similarly to the polyethers, such as an increase in protonated species, but its signal gain was rather small compared to that of other polyethers. By contrast, MALDI-2 of PS was improved in sensitivity by several orders of magnitude, even without the addition of AgTFA. PS preferentially formed radical ions, which we attribute to direct REMPI. This gain in sensitivity makes MALDI-2 a promising technique not only for studying PS MNPs in biological systems but also for analyzing other polymers that absorb at the wavelength of the MALDI-2 laser. Particularly, MS instruments equipped with tunable lasers might broaden the applicability of MALDI-2 to even more polymer classes. Our results pave the way for future studies with MALDI-2 MSI to study the distribution and toxic effects of MNPs in biological samples.

## Supporting Information Available

Additional experimental details, results, and images in support of findings reported in this manuscript (PDF)
