# Supplementary material for: MALDI-2 Mass Spectrometry for Synthetic Polymer Analysis
Source: Macromolecules. 2023 Sep 22;56(19):7729–36. doi: 10.1021/acs.macromol.3c01401 (PMC10569092; doi:10.1021/acs.macromol.3c01401)
Supplement: Supplementary file 1 — ma3c01401_si_001.pdf [file ma3c01401_si_001.pdf]

Supporting information

MALDI-2 mass spectrometry for synthetic polymer analysis

Lidia Molina-Millán[1], Aljoscha Körber[1], Bryn Flinders,[1] Berta Cillero-Pastor[1,2], Eva Cuypers[1], and Ron M.A. Heeren[1]

[1] The Maastricht MultiModal Molecular Imaging Institute (M4i), Division of Imaging Mass Spectrometry, Maastricht University, Universiteitssingel 50, 6229 ER Maastricht, The Netherlands

[2] The MERLN Institute for Technology-Inspired Regenerative Medicine, Department of Cell Biology -Inspired Tissue Engineering, Maastricht University, Universiteitssingel 40, 6229 ER Maastricht, The Netherlands

* Corresponding author: r.heeren@maastrichtuniversity.nl

Table of Contents

Table S1. Experimental *m/z* and intensity values for $[M+H]^+$ species of PEG… ………….........S3

Table S2. Experimental *m/z* and intensity values for $[M+Na]^+$ species of PEG………… ….......S4

Figure S1. MALDI MSI images of PEG at different degrees of polymerization… … … … … …S5

Figure S2. MALDI and MALDI-2 spectra of PPG, nylon-6, PTHF1000, and PTHF1400….S6

Figure S3. Magnification of Figure S2 in the $[M+H]^+$ species… … … … … … … … … … … … …S7

Figure S4. MALDI-2/MALDI ion yields with the degree of polymerization… … … … … ….S8

Figure S5. Kinetics influence in MALDI-2 detection of polymers… … … … … … … … … ….....S9

Figure S6. Effect of the delay time of MALDI-2 laser in ion efficiencies of PPG… …........S10

Figure S7. Effect of the delay time of MALDI-2 laser in ion efficiencies of PPG… …........S11

Figure S8. UV-Vis spectra of PS standard… … … … … … … … … … … … … … … …. … … … …S12

Figure S9. Isotopic distribution of PS confirming $[M+H]^+$ species… … … … … … … … … … …S12

Figure S10. Effect of a second laser ionization step on PEG and PS… … … … … … … S13

Figure S11. MALDI and MALDI-2 spectra of PBT… … … … … … … … … … … … … … … … ….S14

Figure S12. UV-Vis spectra of PBT standard… … … … … … … … …... … … … … … … … ….S15

Table S1. Experimental *m/z* values for $[M+H]^+$ species of PEG 1500 standard obtained with MALDI-2 (left) and MALDI (right). The corresponding peak intensities, elemental composition and mass error (ppm) are shown for each of the values. The mass error for MALDI data are larger because mass calibration was performed only before MALDI-2 acquisition. The MALDI data could be recalibrated internally to decrease mass error.

**MALDI-2**				**MALDI**			
Experimental m/z	Intensity (a.u.)	Ion	Mass error (ppm)	Experimental m/z	Intensity (a.u.)	Ion	Mass error (ppm)
987.59	84.50	$[H(C_2H_4O)_{22}OH+H]^+$	3.3	987.64	12.94	$[H(C_2H_4O)_{22}OH+H]^+$	46.7
1031.62	138.63	$[H(C_2H_4O)_{23}OH+H]^+$	2.5	1031.67	17.22	$[H(C_2H_4O)_{23}OH+H]^+$	47.5
1075.64	251.78	$[H(C_2H_4O)_{24}OH+H]^+$	3.7	1075.70	22.08	$[H(C_2H_4O)_{24}OH+H]^+$	46.3
1119.67	397.69	$[H(C_2H_4O)_{25}OH+H]^+$	3.3	1119.73	28.73	$[H(C_2H_4O)_{25}OH+H]^+$	46.7
1163.69	382.18	$[H(C_2H_4O)_{26}OH+H]^+$	5.6	1163.76	38.36	$[H(C_2H_4O)_{26}OH+H]^+$	49.4
1207.72	473.45	$[H(C_2H_4O)_{27}OH+H]^+$	5.5	1207.78	38.88	$[H(C_2H_4O)_{27}OH+H]^+$	44.5
1251.75	580.36	$[H(C_2H_4O)_{28}OH+H]^+$	5.7	1251.81	55.30	$[H(C_2H_4O)_{28}OH+H]^+$	49.3
1295.77	1174.00	$[H(C_2H_4O)_{29}OH+H]^+$	3.0	1295.84	59.11	$[H(C_2H_4O)_{29}OH+H]^+$	47.0
1339.80	924.27	$[H(C_2H_4O)_{30}OH+H]^+$	5.2	1339.87	54.91	$[H(C_2H_4O)_{30}OH+H]^+$	49.8
1383.83	1357.69	$[H(C_2H_4O)_{31}OH+H]^+$	2.1	1383.90	65.01	$[H(C_2H_4O)_{31}OH+H]^+$	47.9
1427.86	1277.07	$[H(C_2H_4O)_{32}OH+H]^+$	1.3	1427.93	50.88	$[H(C_2H_4O)_{32}OH+H]^+$	48.7
1471.88	1327.13	$[H(C_2H_4O)_{33}OH+H]^+$	4.3	1471.95	45.86	$[H(C_2H_4O)_{33}OH+H]^+$	45.7
1515.91	1318.18	$[H(C_2H_4O)_{34}OH+H]^+$	2.2	1515.98	38.13	$[H(C_2H_4O)_{34}OH+H]^+$	47.8
1559.93	892.57	$[H(C_2H_4O)_{35}OH+H]^+$	1.3	1560.01	34.91	$[H(C_2H_4O)_{35}OH+H]^+$	48.7
1603.96	914.34	$[H(C_2H_4O)_{36}OH+H]^+$	3.5	1604.04	27.98	$[H(C_2H_4O)_{36}OH+H]^+$	46.5
1647.99	632.98	$[H(C_2H_4O)_{37}OH+H]^+$	2.0	1647.94	24.44	$[H(C_2H_4O)_{37}OH+H]^+$	27.0
1692.01	409.41	$[H(C_2H_4O)_{38}OH+H]^+$	1.6	1691.97	22.05	$[H(C_2H_4O)_{38}OH+H]^+$	26.6
1736.03	336.90	$[H(C_2H_4O)_{39}OH+H]^+$	3.8	1736.01	17.90	$[H(C_2H_4O)_{39}OH+H]^+$	18.8
1780.06	225.59	$[H(C_2H_4O)_{40}OH+H]^+$	2.8	1780.03	16.09	$[H(C_2H_4O)_{40}OH+H]^+$	22.8
1824.08	124.76	$[H(C_2H_4O)_{41}OH+H]^+$	4.8	1824.06	12.42	$[H(C_2H_4O)_{41}OH+H]^+$	19.8
1868.11	74.14	$[H(C_2H_4O)_{42}OH+H]^+$	4.1	1868.09	11.41	$[H(C_2H_4O)_{42}OH+H]^+$	14.1

Table S2. Experimental m/z values for $[M+Na]^+$ species of PEG 1500 standard obtained with MALDI-2 (left) and MALDI (right). The corresponding peak intensities, elemental composition and mass error (ppm) are shown for each of the values. The mass error for MALDI data are larger because mass calibration was performed only before MALDI-2 acquisition. The MALDI data could be recalibrated internally to decrease mass error.

**MALDI-2**				**MALDI**			
Experimental m/z	Intensity (a.u.)	Ion	Mass error (ppm)	Experimental m/z	Intensity (a.u.)	Ion	Mass error (ppm)
481.26	19.96	$[H(C_2H_4O)_{10}OH+Na]^+$	7.0	481.29	21.77	$[H(C_2H_4O)_{10}OH+Na]^+$	48.0
525.29	21.68	$[H(C_2H_4O)_{11}OH+Na]^+$	2.4	525.31	23.07	$[H(C_2H_4O)_{11}OH+Na]^+$	47.6
569.31	27.08	$[H(C_2H_4O)_{12}OH+Na]^+$	3.2	569.34	35.00	$[H(C_2H_4O)_{12}OH+Na]^+$	46.8
613.34	46.95	$[H(C_2H_4O)_{13}OH+Na]^+$	5.9	613.37	36.33	$[H(C_2H_4O)_{13}OH+Na]^+$	44.1
657.36	34.24	$[H(C_2H_4O)_{14}OH+Na]^+$	7.7	657.40	39.00	$[H(C_2H_4O)_{14}OH+Na]^+$	47.3
701.39	44.55	$[H(C_2H_4O)_{15}OH+Na]^+$	4.0	701.43	51.01	$[H(C_2H_4O)_{15}OH+Na]^+$	46.0
745.41	52.59	$[H(C_2H_4O)_{16}OH+Na]^+$	7.5	745.46	61.79	$[H(C_2H_4O)_{16}OH+Na]^+$	47.5
789.44	62.70	$[H(C_2H_4O)_{17}OH+Na]^+$	6.5	789.48	54.72	$[H(C_2H_4O)_{17}OH+Na]^+$	43.5
833.47	107.70	$[H(C_2H_4O)_{18}OH+Na]^+$	5.5	833.51	73.29	$[H(C_2H_4O)_{18}OH+Na]^+$	44.5
877.49	154.59	$[H(C_2H_4O)_{19}OH+Na]^+$	5.4	877.54	114.42	$[H(C_2H_4O)_{19}OH+Na]^+$	44.6
921.52	190.98	$[H(C_2H_4O)_{20}OH+Na]^+$	4.9	921.57	164.65	$[H(C_2H_4O)_{20}OH+Na]^+$	45.1
965.55	351.17	$[H(C_2H_4O)_{21}OH+Na]^+$	4.2	965.60	340.03	$[H(C_2H_4O)_{21}OH+Na]^+$	45.8
1009.57	508.85	$[H(C_2H_4O)_{22}OH+Na]^+$	2.3	1009.63	650.35	$[H(C_2H_4O)_{22}OH+Na]^+$	47.7
1053.60	830.40	$[H(C_2H_4O)_{23}OH+Na]^+$	1.8	1053.65	1101.47	$[H(C_2H_4O)_{23}OH+Na]^+$	48.2
1097.63	1810.21	$[H(C_2H_4O)_{24}OH+Na]^+$	3.8	1097.68	1568.20	$[H(C_2H_4O)_{24}OH+Na]^+$	46.2
1141.65	1863.45	$[H(C_2H_4O)_{25}OH+Na]^+$	5.6	1141.71	2619.63	$[H(C_2H_4O)_{25}OH+Na]^+$	49.4
1185.68	3950.19	$[H(C_2H_4O)_{26}OH+Na]^+$	4.1	1185.74	2463.49	$[H(C_2H_4O)_{26}OH+Na]^+$	45.9
1229.70	5321.59	$[H(C_2H_4O)_{27}OH+Na]^+$	3.0	1229.77	5098.94	$[H(C_2H_4O)_{27}OH+Na]^+$	47.0
1273.73	6650.68	$[H(C_2H_4O)_{28}OH+Na]^+$	4.2	1273.79	3443.39	$[H(C_2H_4O)_{28}OH+Na]^+$	45.8
1317.75	6853.20	$[H(C_2H_4O)_{29}OH+Na]^+$	5.0	1317.83	7000.34	$[H(C_2H_4O)_{29}OH+Na]^+$	50.0
1361.78	8815.38	$[H(C_2H_4O)_{30}OH+Na]^+$	4.0	1361.85	4691.47	$[H(C_2H_4O)_{30}OH+Na]^+$	46.0
1405.81	9220.63	$[H(C_2H_4O)_{31}OH+Na]^+$	2.2	1405.88	8980.72	$[H(C_2H_4O)_{31}OH+Na]^+$	47.8
1449.83	9269.37	$[H(C_2H_4O)_{32}OH+Na]^+$	4.1	1449.91	4867.30	$[H(C_2H_4O)_{32}OH+Na]^+$	50.9
1493.86	8986.67	$[H(C_2H_4O)_{33}OH+Na]^+$	3.5	1493.93	6642.68	$[H(C_2H_4O)_{33}OH+Na]^+$	46.5
1537.89	8406.95	$[H(C_2H_4O)_{34}OH+Na]^+$	3.9	1537.97	4250.33	$[H(C_2H_4O)_{34}OH+Na]^+$	51.1
1581.91	6768.60	$[H(C_2H_4O)_{35}OH+Na]^+$	4.5	1582.00	5765.68	$[H(C_2H_4O)_{35}OH+Na]^+$	50.5
1625.94	3613.69	$[H(C_2H_4O)_{36}OH+Na]^+$	5.2	1626.02	5588.03	$[H(C_2H_4O)_{36}OH+Na]^+$	49.8
1669.97	4883.27	$[H(C_2H_4O)_{37}OH+Na]^+$	2.5	1670.05	4580.39	$[H(C_2H_4O)_{37}OH+Na]^+$	47.5
1713.99	3438.55	$[H(C_2H_4O)_{38}OH+Na]^+$	4.5	1714.08	3263.82	$[H(C_2H_4O)_{38}OH+Na]^+$	50.5
1758.02	2576.10	$[H(C_2H_4O)_{39}OH+Na]^+$	1.5	1758.11	2572.56	$[H(C_2H_4O)_{39}OH+Na]^+$	48.5
1802.05	1595.17	$[H(C_2H_4O)_{40}OH+Na]^+$	1.1	1802.14	1713.73	$[H(C_2H_4O)_{40}OH+Na]^+$	48.9
1846.07	1200.66	$[H(C_2H_4O)_{41}OH+Na]^+$	3.7	1846.17	613.33	$[H(C_2H_4O)_{41}OH+Na]^+$	51.3
1890.10	733.05	$[H(C_2H_4O)_{42}OH+Na]^+$	2.4	1890.19	633.15	$[H(C_2H_4O)_{42}OH+Na]^+$	47.6
1934.12	417.62	$[H(C_2H_4O)_{43}OH+Na]^+$	3.3	1934.22	291.20	$[H(C_2H_4O)_{43}OH+Na]^+$	46.7
1978.15	159.07	$[H(C_2H_4O)_{44}OH+Na]^+$	1.0	1978.25	226.25	$[H(C_2H_4O)_{44}OH+Na]^+$	49.0
2022.17	110.44	$[H(C_2H_4O)_{45}OH+Na]^+$	3.2	2022.27	89.63	$[H(C_2H_4O)_{45}OH+Na]^+$	46.8

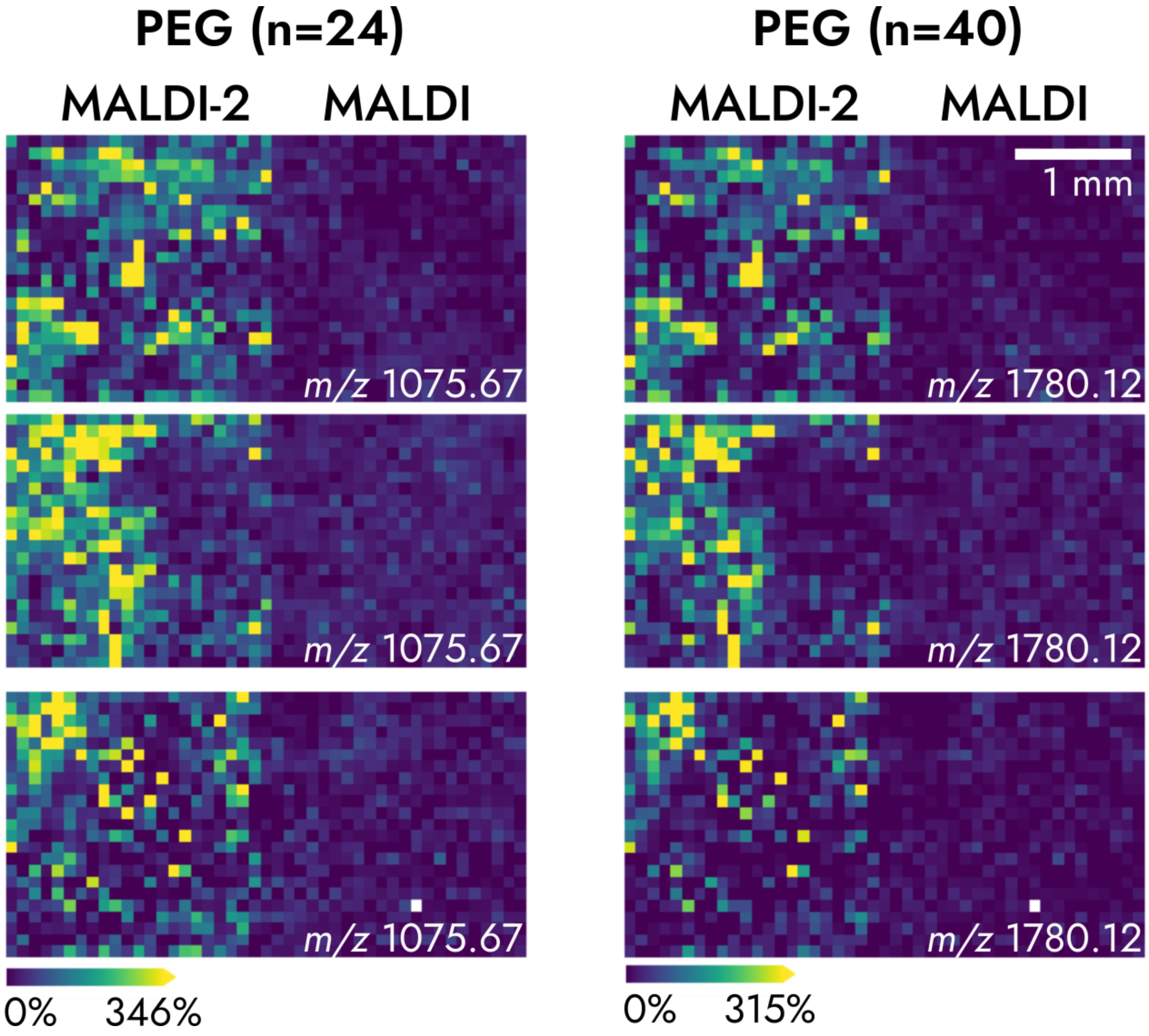

Figure S1. MALDI MSI images of PEG [M+H]$^+$ at *m/z* 1075.67 (n=24) and *m/z* 1780.12 (n=40). The images illustrate that MALDI-2 signals are higher for all degrees of polymerization of PEG.

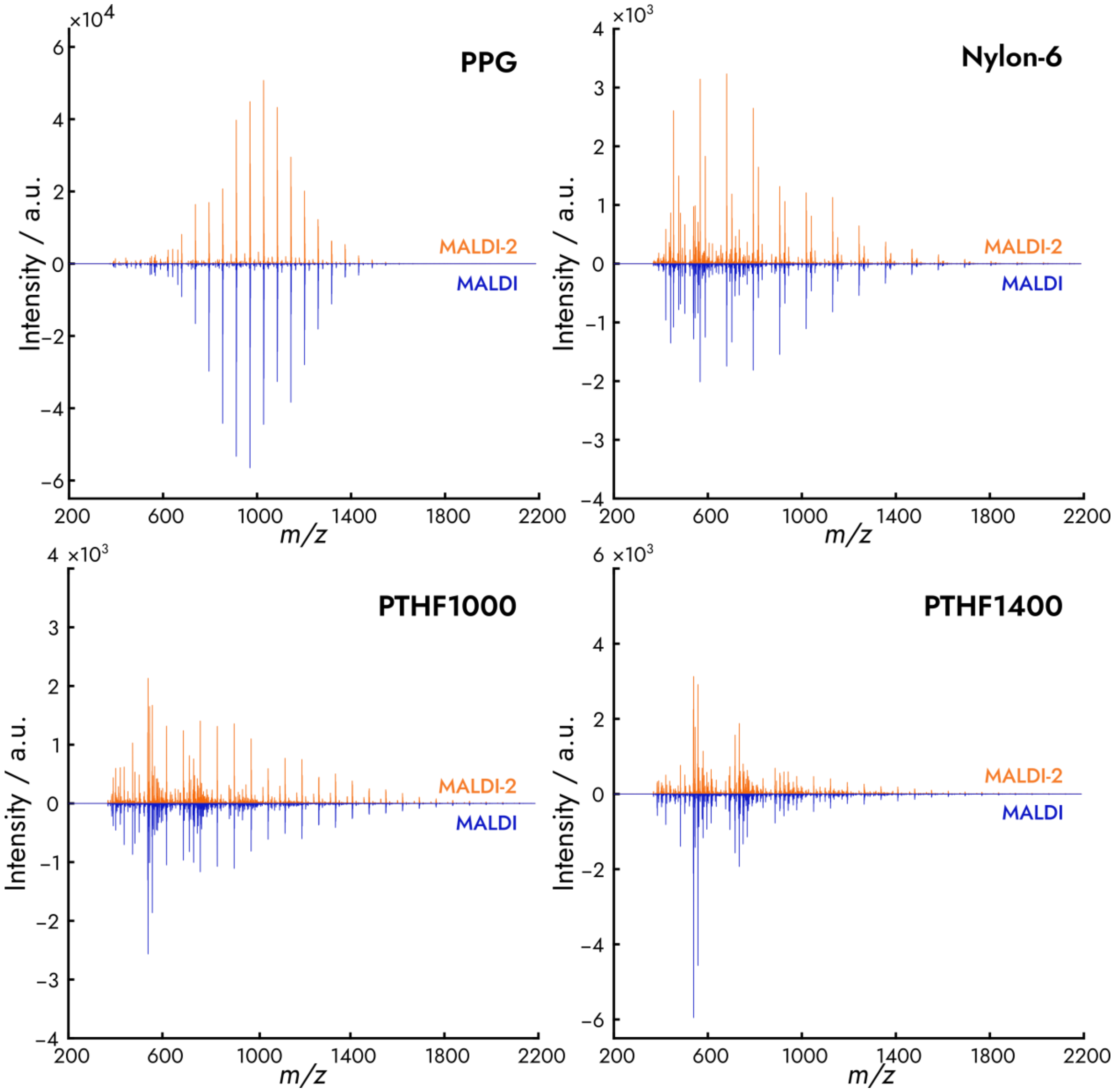

Figure S2. MALDI-2 (top, orange) and MALDI (blue, bottom) spectra of PPG, nylon-6, PTHF1000, and PTHF1400. PPG behaves very similarly to PEG, with both MALDI-2 and MALDI spectra being dominated by sodiated ions. The protonated species of PPG increase in signal up to 140-fold (see **Figure S3**). MALDI-2 of nylon-6 yielded only a comparatively minor signal enhancement, which we attribute to its higher molecular weight distribution. PTHF1000 and PTHF1400 were detected with lower ion yields than PEG and PPG. Still, we could see in increase in the protonated species with MALDI-2 (see **Figure S3**).

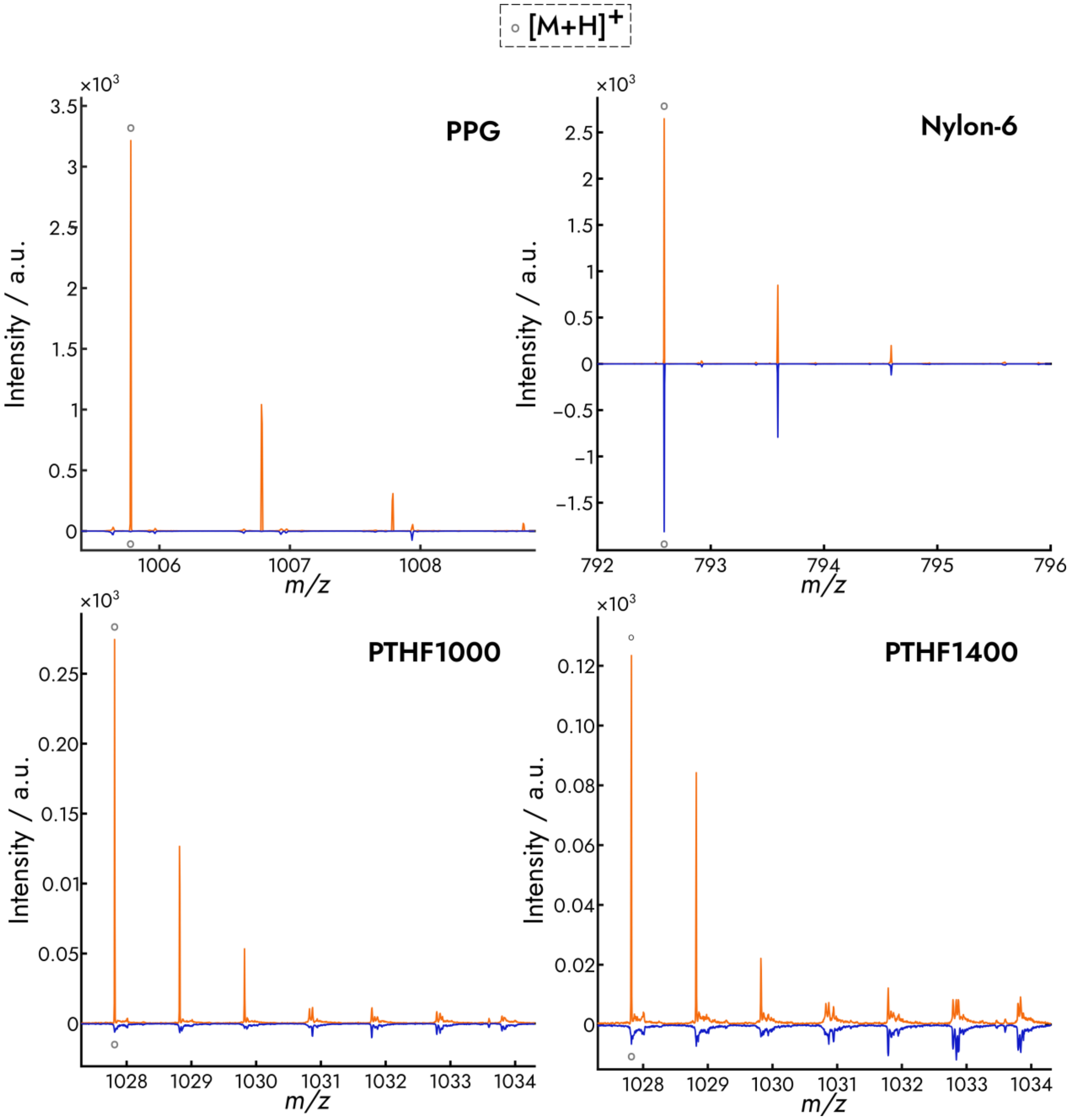

Figure S3. Magnification into the isotopic fine structure of [M+H]$^+$ ions shown in **Figure S2**. Here, we demonstrate an increase in the protonated species with MALDI-2 for all polymers tested. MALDI-2 (top, orange) and MALDI (bottom, blue) mass spectra of PPG, nylon-6, PTHF1000, and PTHF1400.

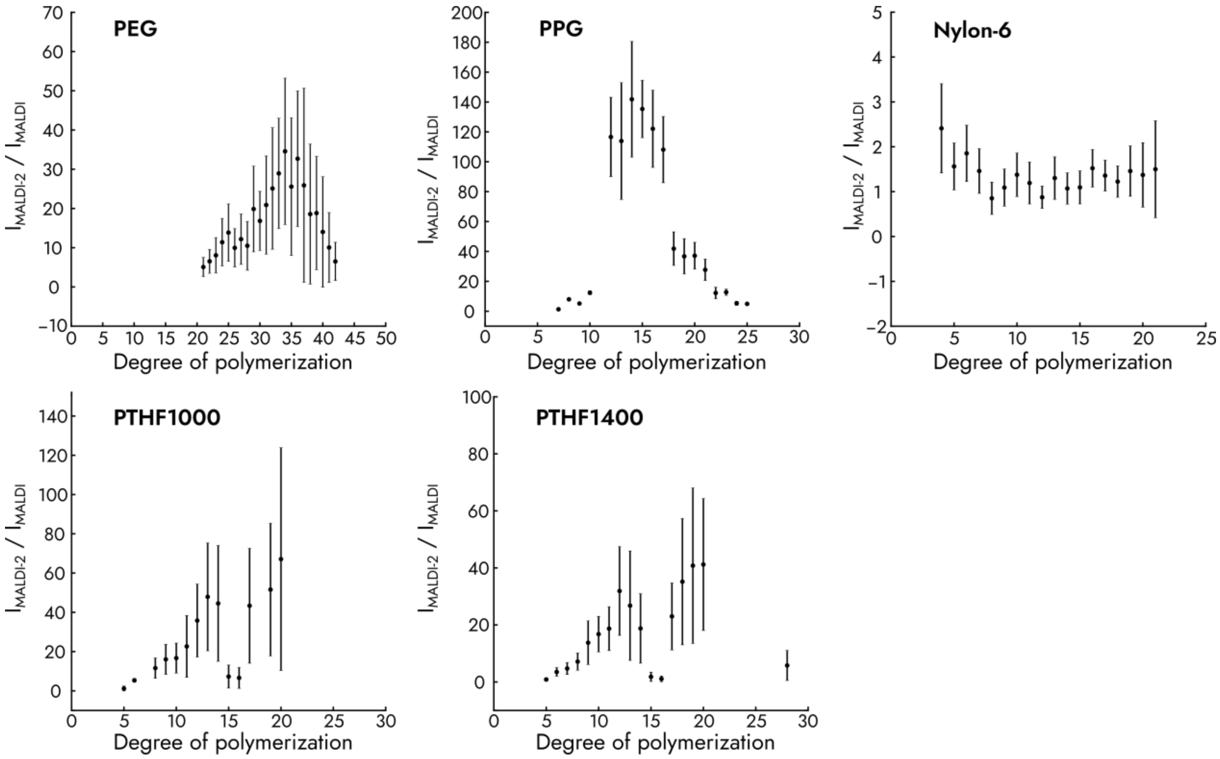

Figure S4. MALDI-2/MALDI ion yield ratio of $[M+H]^+$ species plotted against degree of polymerization (n) for different polymer standards. MALDI-2/MALDI ratios of the performed experiments (n = 5) were calculated with the height intensities of the identified peaks. Error bars correspond to the standard deviation. Only three replicates were considered for PEG (n=3) since the other two replicates exhibited peak shifts and peak splitting artefacts that we attribute to voltage modifications in the timsTOF fleX. The trend for PEG shows a maximum close to the middle of the molecular weight distribution, which could indicate that the ionization yield in MALDI-2 is higher for higher abundant species due to faster kinetics (see **Figure S5**). However, we do not observe such behaviour for PPG, where the maximum ionization ratio is located at a lower *m/z* than the maximum of its molecular weight distribution (see **Figure S5**). This indicates that PPG might be influenced by other factors than abundance. Nonetheless, both PEG and PPG show an increase in the protonated species with MALDI-2 for all the degrees of polymerization. Nylon-6 ion yield ratios remain constant with the degree of polymerization. PTHF1000 and PTHF1400 present lower ion yields and higher variability in the data which hinders the visualization of the behaviour of these polymers with the degree of polymerization. The behaviour of nylon-6 and PTHF remains inconclusive with these data.

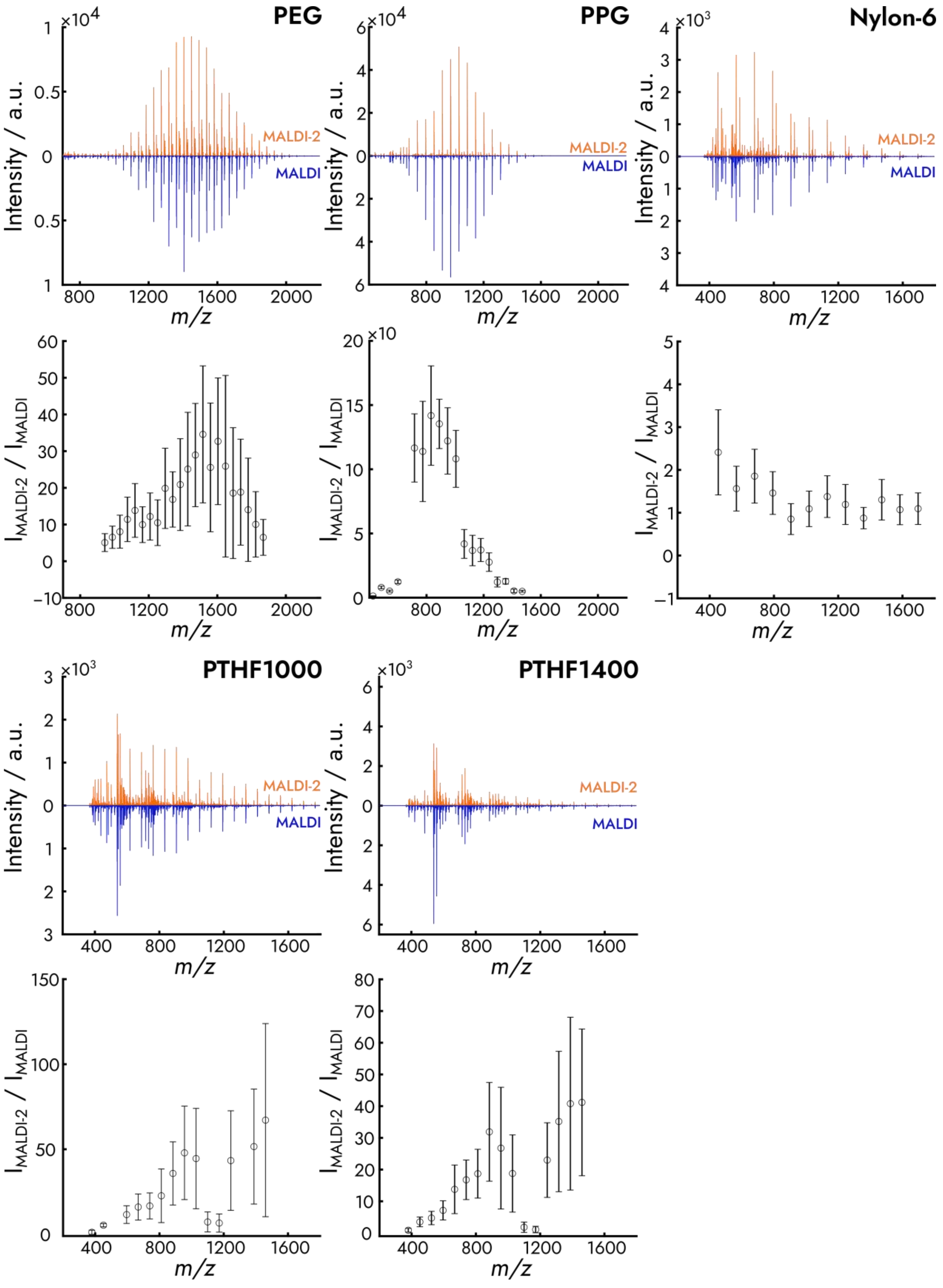

Figure S5. Kinetics study of different polymer standards. MALDI-2 (orange) and MALDI (blue) spectra are compared with graphs shown in **Figure S4**. The center of each polymer distribution does not match with the largest signal enhancement.

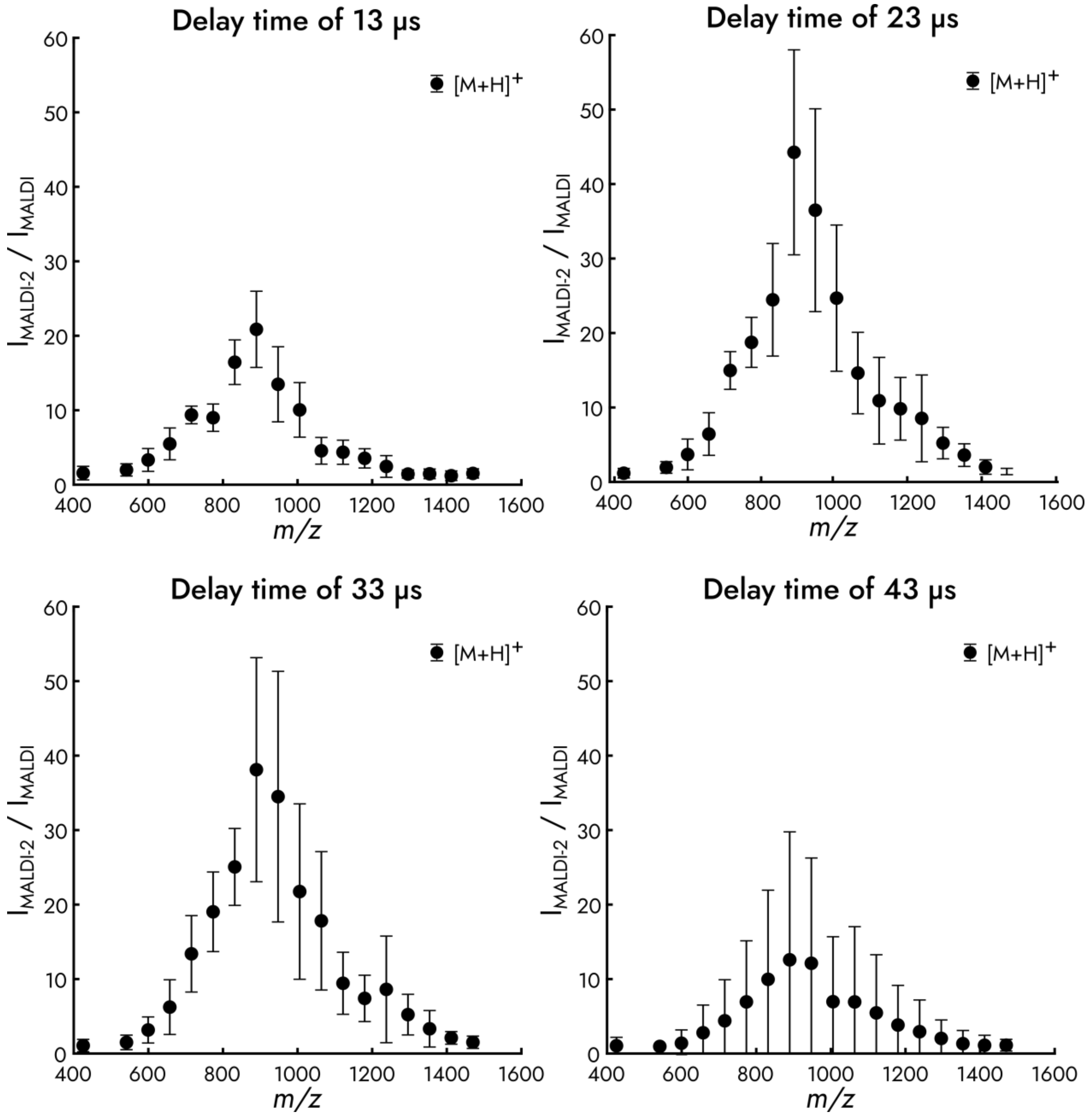

Figure S6. Delay time (t_{delay}) experiments performed with PPG sprayed onto an ITO-slide. We observed an increase in the ion yield ratios with the delay time meaning that the overall ion efficiency also increased. We concluded that delay times between 23 and 33 µs were the most optimal for polymer analysis with MALDI-2. For t_{delay}<13 µs and t_{delay}>43 µs, we observed a decrease in ionization efficiency (data not shown).

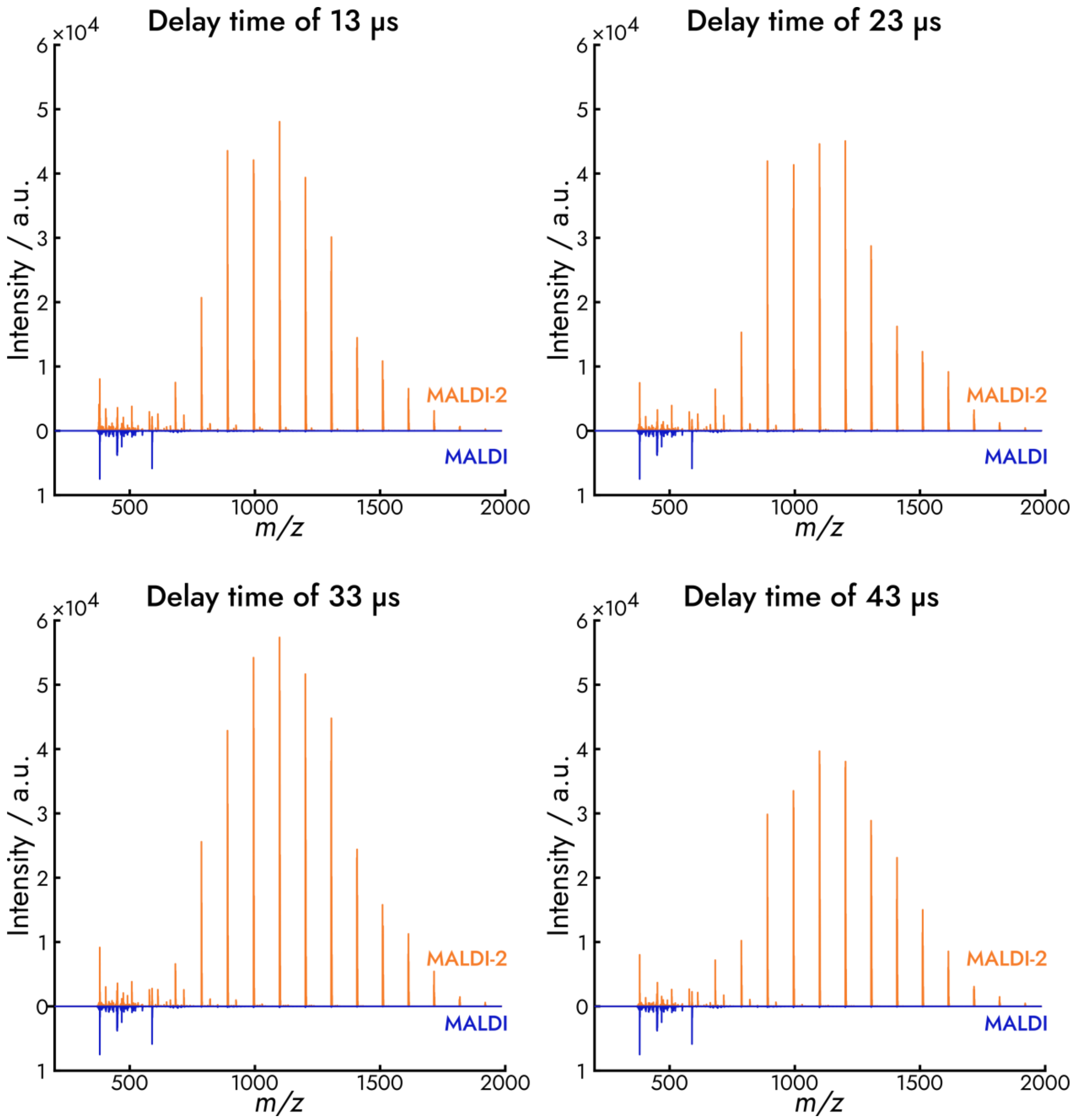

Figure S7. Delay time (t_{delay}) experiments performed with PS sprayed with DT onto an ITO-slide. PS is presented differently in comparison to the other polymers since no polymer signals were obtained with MALDI. Hence, ion yield ratios could not be calculated for PS. For PS, we observed only minor changes in ion yield with delay time. This finding supports our theory that PS is ionized with direct 1+1 REMPI, which is less dependent on plume density and gas phase collision rates than MALDI-2.

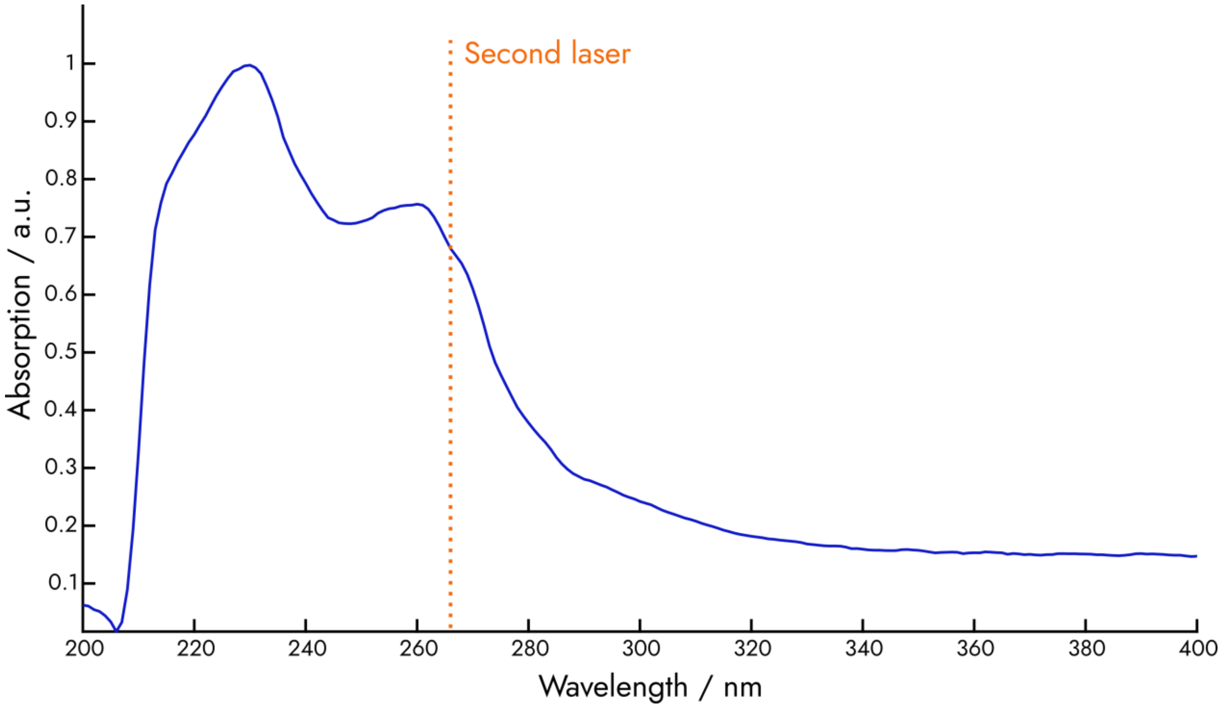

Figure S8. UV-Vis spectrum of PS standard in THF solution featuring broad absorption bands at 230 and 256 nm. At the wavelength of the MALDI-2 laser (266 nm, highlighted in orange), PS absorbs strongly. Thus, it is plausible that upon laser irradiation, PS in the gas phase can ionize directly via 1+1 REMPI.

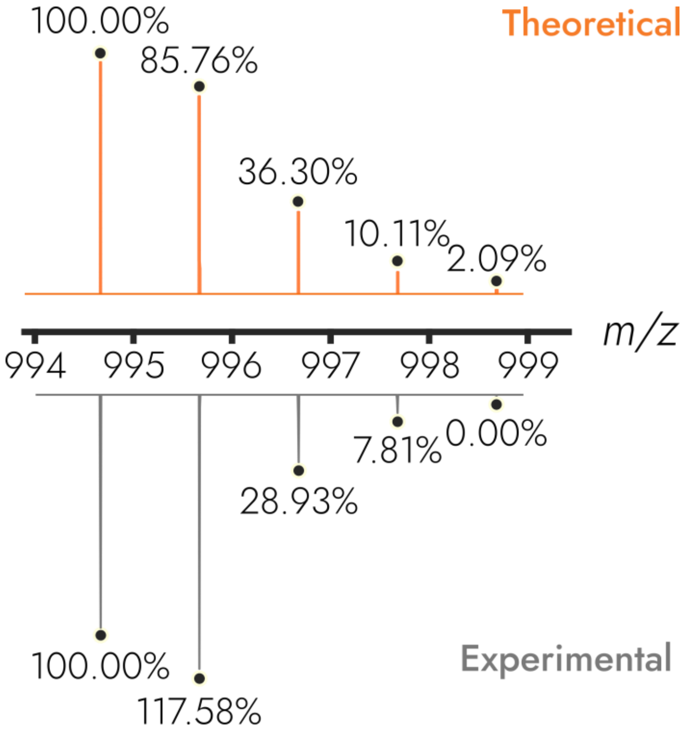

Figure S9. Theoretical (top, orange) and experimental (bottom, grey) isotopic distributions of the PS oligomer $[C_{76}H_{82}]^{+\bullet}$ at *m/z* 994.64. The higher abundance in $[C_{75}{}^{13}CH_{82}]^{+\bullet}$ (*m/z* 995. 64) reveals the presence of protonated species when analyzing PS with DT matrix and AgTFA with MALDI-2.

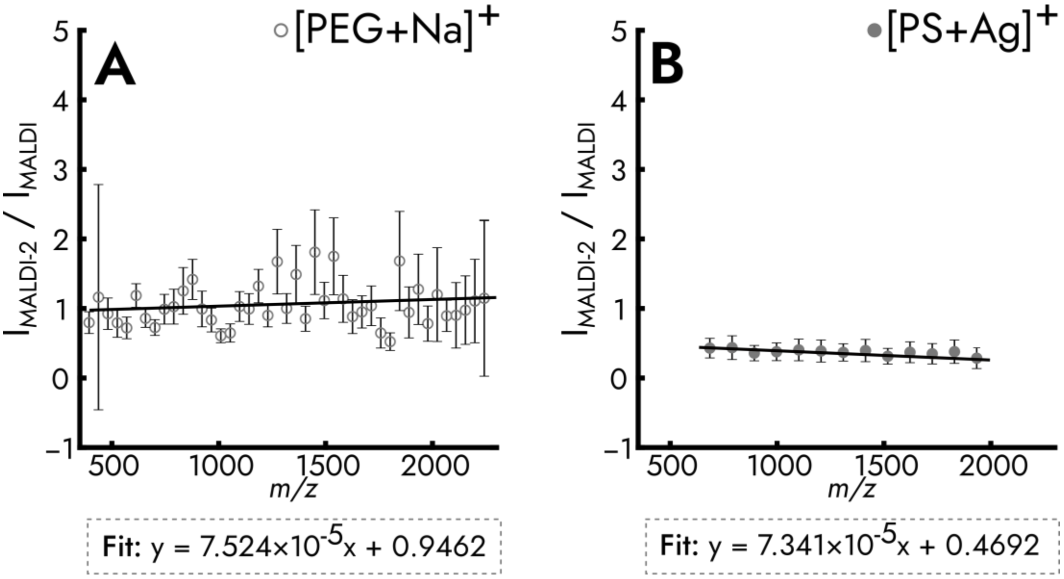

Figure S10. Effect of a second laser ionization step on PEG (**A**) and PS (**B**). The PEG sample solution was sodiated prior to spraying to produce large $[M+Na]^+$ peaks and therefore reduce the influence of statistical fluctuations. Lines in both graphs correspond to fits of the data points presented. In the case of PEG (**A**) the intensity of the sodiated species fluctuates around one, indicating that sodiated PEG cations are not significantly affected by MALDI-2. For PS (**B**) however, the intensities of the silver adduct ions decrease with MALDI-2 to about half of their initial value. We interpret these differences such that PEG ionizes via the postulated MALDI-2 mechanism, which does not affect sodiated ions. PS on the contrary absorbs strongly at 266 nm, allowing not only for direct 1+1 REMPI but also for resonant photo-fragmentation, which, together with the observance of very weak fragment peaks in the mass spectra (data not shown), could explain a decrease in silver adduct ions. The differences observed in the size of the error bars can be explained by the specific deposition behavior of the polymers in the ITO-slide as well as by the fact that REMPI depends on fewer experimental parameters than MALDI-2, for example plume density, pressure, and the collision-cross section of the matrix.

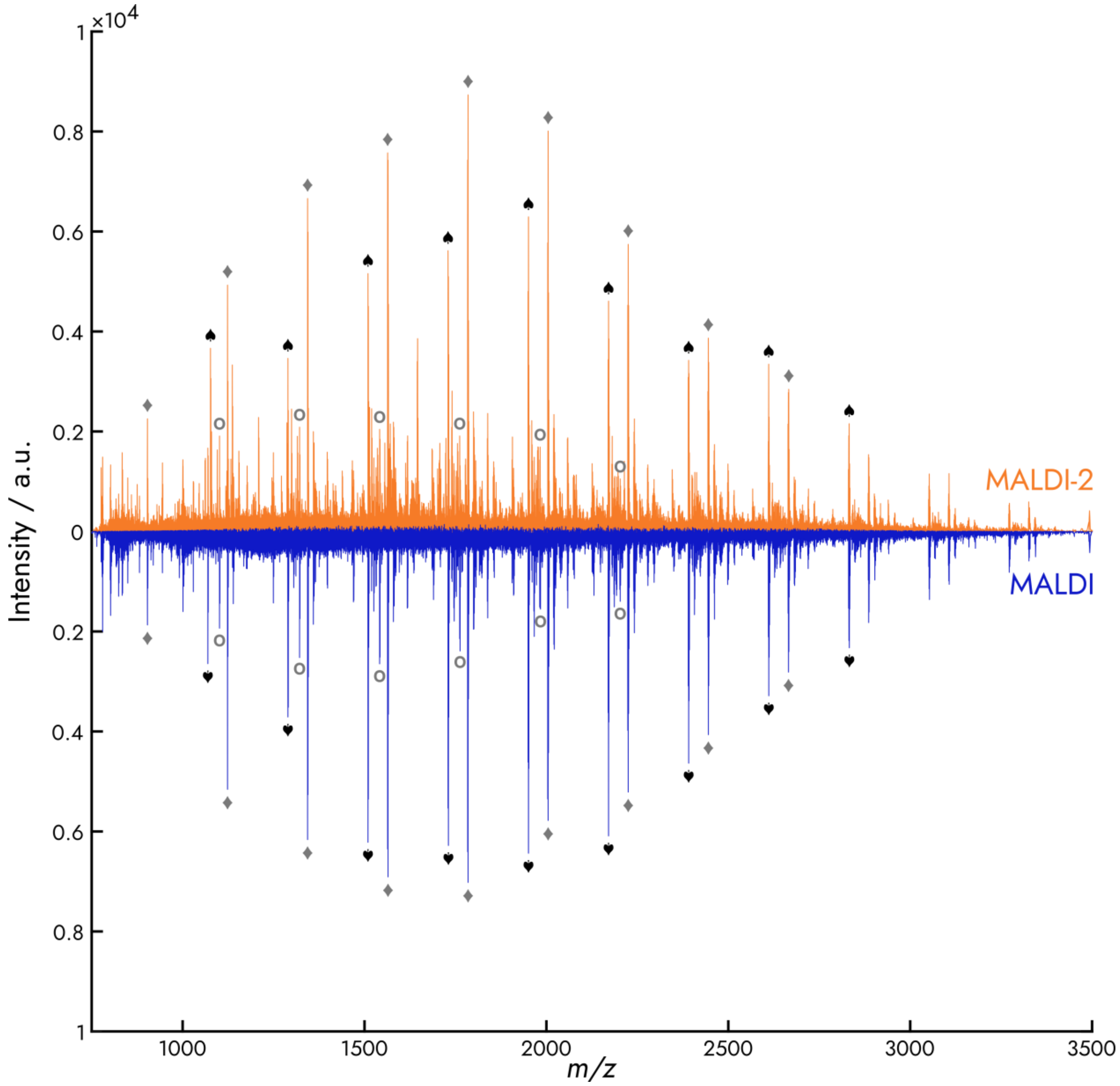

Figure S11. MALDI-2 (top, orange) and MALDI (bottom, blue) spectra of PBT with the general structure R_1-[O-$(CH_2)_4$-OOC-C_6H_4CO]$_n$-R_2,in short R_1-[PBT]$_n$-R_2. Tentatively assigned peaks include protonated (o) and sodiated (♦) peaks of cyclic PBT, respectively [HOOC-C_6H_4-CO-(PBT)$_n$-O-$(CH_2)_2$-CH=CH_2 + H]$^+$ and [HOOC-C_6H_4-CO-(PBT)$_n$-O-$(CH_2)_2$-CH=CH_2 + Na]$^+$, as well as [HOOC-C_6H_4-CO-(PBT)$_n$-OH + Na]$^+$ (♠). For PBT, MALDI-2 does not cause an increase in radical ion yield, which we attribute to the fact that PBT does not absorb at the wavelength of our MALDI-2 laser (266 nm, see UV spectrum **Figure S12**).

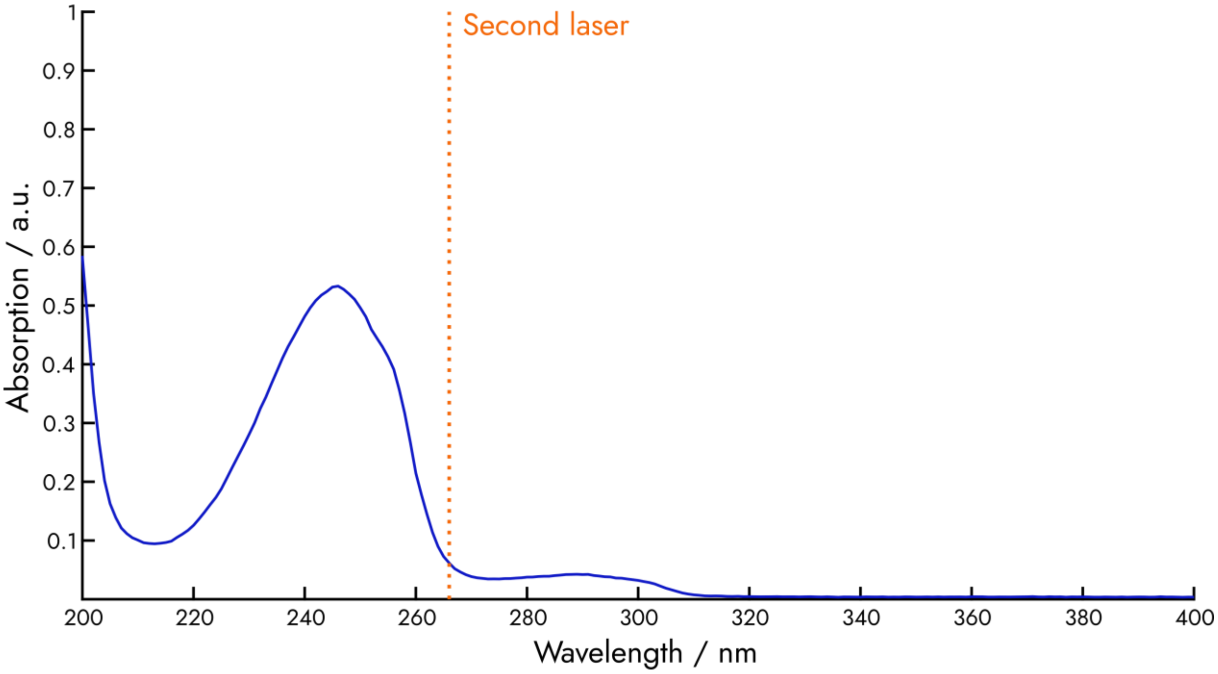

Figure S12. UV-Vis spectrum of PBT standard in HFIP solution featuring a single absorption band 246 nm. At the wavelength of the MALDI-2 laser (266 nm, indicated in orange), PBT absorption is negligible.
